# Chronic lymphocytic leukemia-associated paraneoplastic pemphigus: potential cause and therapeutic strategies

**DOI:** 10.1038/s41598-020-73131-y

**Published:** 2020-10-01

**Authors:** Lei Cao, Fei Wang, Xin-Yi Du, Hua-Yuan Zhu, Li Wang, Wei Xu, Jian-Yong Li, Lei Fan

**Affiliations:** 1 Department of Hematology, The First Affiliated Hospital of Nanjing Medical University, Jiangsu Province Hospital, Nanjing, 210029 China; 2 Key Laboratory of Hematology of Nanjing Medical University, Nanjing, 210029 China; 3 Collaborative Innovation Center for Cancer Personalized Medicine, Nanjing, 210029 China

**Keywords:** Tumour immunology, Leukaemia

## Abstract

Paraneoplastic pemphigus (PNP) is a severe autoimmune syndrome commonly triggered by neoplasms. The prognosis of CLL-associated PNP is dismal due to its refractory course and secondary infection and no standard treatment was recommended. We retrospectively reported six CLL with PNP cases from 842 cases of CLL including diagnosis, treatment and prognosis. The median time between the initial of CLL to PNP was 36 months while the median overall survival from the diagnosis of PNP was 26 months. And three cases died of lung infection while 5 developed pulmonary symptoms. And 5 cases received fludarabine-based chemotherapy before developing PNP, which suggesting fludarabine was one of potential causes of PNP. For the treatment, five patients were rescued by combined regimens including rituximab, methylprednisolone, immunoglobulin, fresh frozen plasma and the last received ibrutinib combined with short-term prednisone. Fludarabine-based regimen may be one of the potential causes of PNP. The combined regimen might shed a new light, while ibrutinib is a promising drug for CLL with PNP, but needs much more evidence. PNP should be carefully treated to guide early diagnosis and intervention for a better prognosis.

## Introduction

Paraneoplastic pemphigus (PNP), as a severe autoimmune disease, is categorized by autoantibodies like IgG that injures desmoplakins, desmogleins, desmocollins and presenting a poor prognosis with a high mortality due to its resistance to routine immunosuppression, immunomodulation, or plasmapheresis treatments^1^. PNP can manifest clinically in diverse painful stomatitis, polymorphous skin eruption, or the presence of antibodies against desmogleins (Dsg) 1 and 3, envoplakin, and periplakin^2,3^. According to the research reported to the US Food and Drug Administration, a total of 12 in 100,000 cases of non-Hodgkin’s lymphoma (NHL) and chronic lymphocytic leukemia (CLL) were found to be complicated by PNP^4^. The relatively more associated disorders were NHL for 38.6%, and other hematologic tumors or disorders were Castleman’s disease, CLL, thymoma, Waldenström’s macroglobulinemia^5^. The notable correlation between PNP and lymphoproliferative diseases (LPD) might indicate some immune inefficiency of lymphocytes resulting in PNP, and reversely might offer some clews for LPD development.

CLL is a most prevalent adult lymphoid malignance in western hemisphere accounting for 30% of all leukemia, but relatively rare in eastern countries^6^. It often occurs in the elderly people with male-predominance^7^. About 5–10% of such patients might develop autoimmune complications at any stage in the process of CLL. The mechanisms between CLL and autoimmune complications have not been fully uncovered despite of unremitting efforts. Compared with the hematologic autoimmune complications, non-hematologic autoimmune complications such as PNP, glomerulonephritis, C1 esterase deficiency and pernicious anemia are relatively rare^2,8^.

Although about 25% of CLL patients might endure mild cutaneous lesions caused by autoimmune diseases or over-immune reaction of CLL^9^, but PNP as a severe and life-threatening disease, still draws much more attentions. Here we reported 6 cases of CLL-associated PNP throughout all their course up to May in 2020 and analyzed the clinical and biologic features to explore the potential associations between CLL and PNP.

## Materials and methods

We retrospectively analyzed 842 CLL patients presented in our hospital from 2007 to 2018. The diagnosis of CLL was based on iwCLL criteria including clinical features, blood and bone marrow examination including morphology, immunology, cytogenetics and molecular biology. Specific clonal B lymphocytes expressing CD5, CD19, light chain, CD23 and CD79b were detected by flow cytometry in periphery blood [positive for light chain restriction (either kappa or lambda), CD5, CD23, CD79b, and surface immunoglobulin expression, and low levels of CD20]^10^. Chronic lymphocytic leukemia can be diagnosed if Matutes score reaches 4 points or more. Investigations to diagnose CLL-associated PNP should consist of checking for systemic complications to identify CLL, skin biopsies for histopathological and immunofluorescence studies, and serum immunological studies such as lichenoid or acantholytic changes in pathology, supportive immunofluorescence findings, particular intercellular and basement membrane binding^11^. All patients showed increased lymphocytes in periphery blood with typical morphology and immunophenotype of CLL scored with 4–5 tested by flow cytometry. Immunohistochemistry of Cyclin D1 or fluorescence in situ hybridization (FISH) of t(11;14) was considered routinely in cases with atypical immunophenotype. PNP was diagnosed based on the skin biopsy and immunoprecipitation^11^. All of them were diagnosed as CLL previously and developed PNP during the treatment or follow-up of CLL. The baseline characteristics of the 6 patients with PNP were shown in Table 1.

**Table 1:** Clinical characteristics of 6 CLL patients from the time of PNP diagnosis.

Patient	P1	P2	P3	P4	P5	P6
Age (years old)	48	38	55	81	58	49
Gender	Male	Male	Male	Female	Female	Male
Rai	IV	III	IV	III	II	I
Binet	C	C	C	C	B	B
ALC (× 10^9^/L)	7.7	7.91	41.13	> 100	17.26	37
PLT (× 10^9^/L)	< 100	112	71	169	122	Normal
Hb (g/L)	< 100	94	98	< 100	128	Normal
LDH (140–271 U/L)	146	519	692	310	385	Normal
β2-MG (1.09–2.53 μmol/L)	2.75	16.7	11.10	NA	3.33	Normal
EBV DNA (< 5.00E + 2 copies/mL)	Normal	NA	Normal	Normal	Normal	Normal
ALB (35–52 g/L)	29.4	27.5	43.4	41.1	52.9	Normal
IgG (7.0–16.0 g/L)	NA	28.2	9.1	5.34	6.18	4.75
C3 (0.9–1.8 g/L)	1.16	NA	0.87	1.02	0.68	0.97
CA-125 (< 35 U/mL)	1.2	45.6	40.28	296.5	10.5	Normal
ATM (< 8%)	8/300	Positive	24/300	NA	Negative	NA
17p-deletion (< 5%)	Negative	Positive	Negative	Negative	Negative	Negative
IGHV mutation	Unmutated, IGHV1-69*01, 100%	NA	Unmutated, IGHV3-15*02, 99.2%	Mutated, IGHV3-30*03, 86.86%	Mutated, IGHV3-23*01, 95.49%	NA
Karyotype	NA	46,XY,t(3;11)(q26;q13)[1]/46,XY[9]	NA	NA	47,XX,+12[10]	NA
CLL-IPI	3	7–9	5	2–4	1	1–3
CLLIPI-risk	Low-medium	High	High-medium	Medium	Low	Low-medium
Status	Alive (2015–06, lost)	Dead(2007–10)	Dead(2013–07)	Dead(2017–11)	Alive(2020–05)	Alive(2020–05)
Time of CLL diagnosis	2011-06	2004-09	2002-11	2003-12	2014-10	2016-12
Time of PNP diagnosis (CLL state)	2012-09 (PR)	2006-09 (PD)	2012-06 (PD)	2015-06 (PR)	2018-08 (PR)	2017-11 (PD)
Time interval from CLL to PNP	16 m	25 m	116 m	139 m	47 m	12 m
F-based regimen	Yes	Yes	Yes	Yes	Yes	No
The timing of fludarabine use to the development of PNP	15 m	22 m	107 m	72 m	10 m	NA
Pulmonary symptoms	Exist	Exist	Exist	Exist	No	Exist
OS (PNP)	33 m	14 m	14 m	30 m	22 m	31 m

### Ethics declarations

Appropriate CLL-PNP consents were obtained from all donors prior to specimen collection. The study was approved by the ethics committees of the First Affiliated Hospital of Nanjing Medical University.

### Approval for human experiments

The research was designed in accordance with the Declaration of Helsinki.

### Informed consent

Informed consent was obtained from all individual participants included in the study.

## Results

According to our data, a total of 0.71% incidence of PNP in CLL population could be drawn due to 6 PNP cases in 842 CLL patients. Besides, 4 of the 6 patients in our study were male. All the 6 patients developed PNP after the treatment of CLL and half of them were diagnosed PNP during the partial remission of CLL while the rest of them manifested as disease progression in CLL. The median time from the initial of CLL to the diagnosis of PNP was 36 months (range from 12 to 139 ms). The median overall survival from the diagnosis of PNP to the recent fellow-up or the death date was 26 months (range from 14 to 33 ms). Half of the 6 patients died while 2 cases were alive and 1 lost follow-up in Jun 2015.

In our study, 4 of the 6 patients presented as Binet C stage with decreased platelet count or hemoglobin, among which only patient 1 and 2 received treatment at the time of diagnosis of CLL. Only case 2 (dead) existed the deletion of 17p and ATM while case 4 (dead) and case 5(alive) existed the mutation of immunoglobulin heavy-chain variable region (IGHV) gene, and case 1 presented as IGHV1-69*01 indicating poor outcome. Besides, 5 cases of them received fludarabine-based regimen before the development of PNP except case 6.

Expressions of the IgG and C3 level in periphery blood were shown at a mild reduction level in nearly half of our PNP cases. Lactate dehydrogenase (LDH) was found to be elevated in 4 of the 6 patients, and its relevance was worthy to be distinguished with PNP complicated with CLL. β_2_-Microglobulin (β_2_-MG) in 2 of the 6 patients were found obviously increased while 1 case wasn’t tested. Serum CA-125 was elevated in 3 patients. Albumin was fall below normal level in patient 1 and patient 2 but kept in the normal rang in others. EBV DNA was negative in 5 of the 6 patients, which might indicate EBV took less effect on the development of CLL-associated PNP. Interestingly, 5 of the 6 cases developed various degrees of pulmonary infection after the diagnosis of PNP.

**Figure 1:**
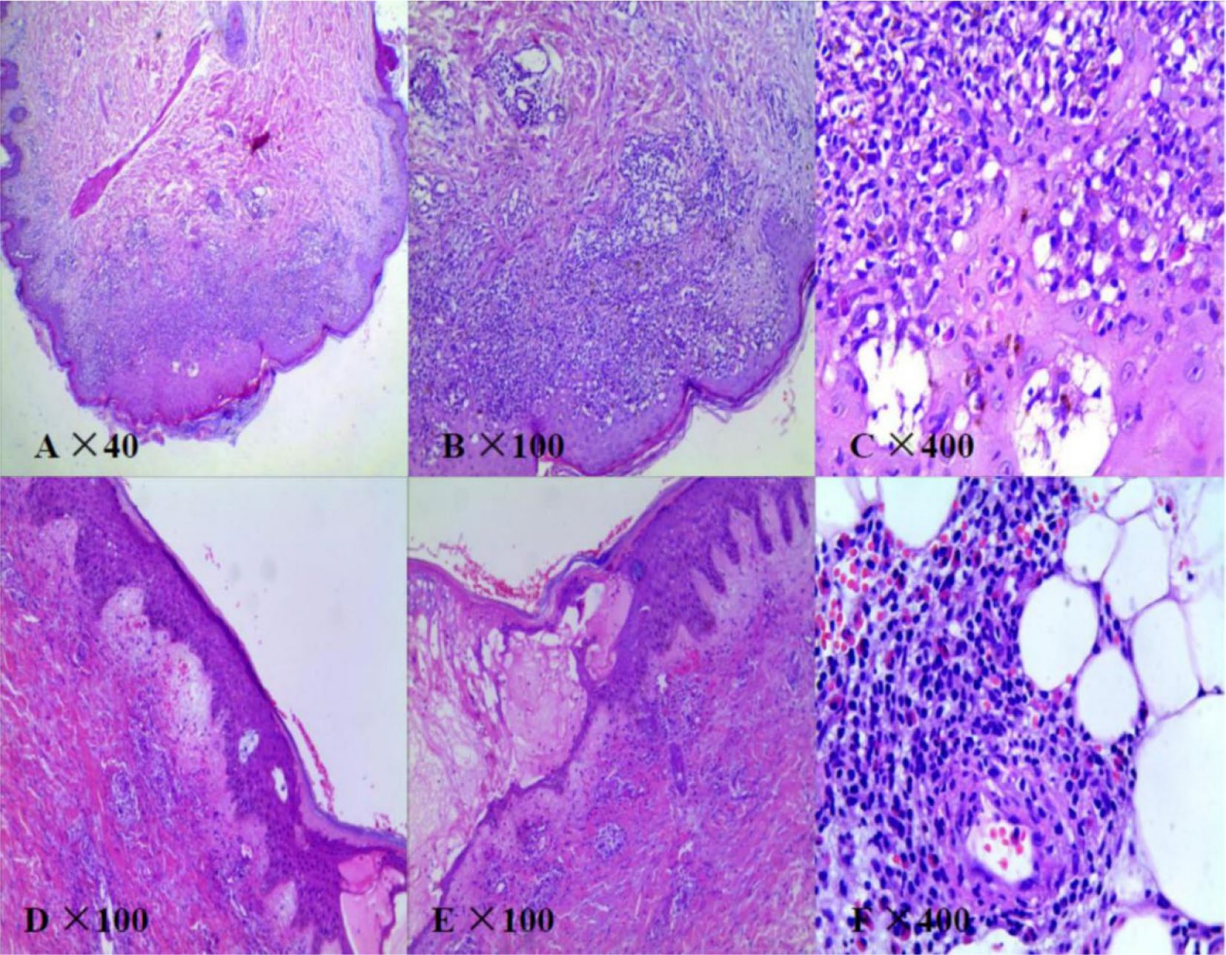
Hematoxylin and eosin (HE) staining of skin lesions (patient 1: (**A**) original magnification × 40, (**B**) original magnification × 100, (**C**) original magnification × 400; patient 4: (**D**) original magnification × 100, (**E**) original magnification × 100, (**F**) original magnification × 400). Cracks and blisters can be easily seen in sliced side of the epidermis. Acantholism released cells and mixed inflammatory cell infiltrated in blisters. Besides hyperkeratosis existed in sliced central, individual keratinocytes or intracellular cell blisters, liquefaction degeneration of basal cells, local formation of small subcutaneous cracks existed in this figure. Diffuse banded lymphocytes infiltrated in dermal superficial lesions, and phagocytic cells were found easily. It was proved to be moss-like dermatitis changes, especial vice-tumor pemphigus.

**Figure 2:**
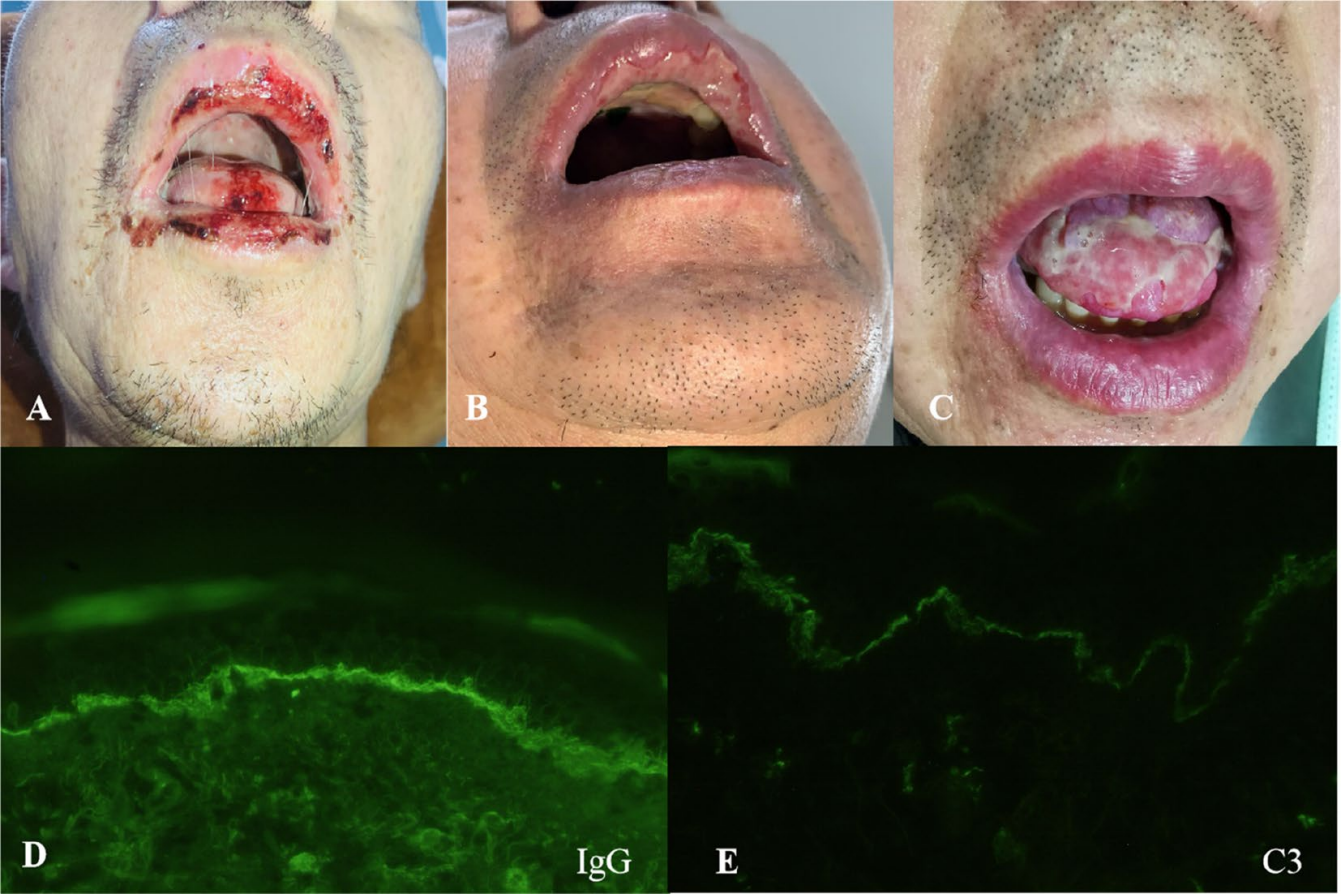
Changes of skin lesions before and after treatment and direct immunofluorescence. (**A**–**C**) Erosive mucositis and ulcers of the lips and oral mucosa before and after treatment (patient 6). (**D**,**E**) IgG and C3 were deposited linearly in the basement membrane respectively (patient 1).

Patient 1 as a 48-year-old male was diagnosed of CLL owing to his lymphocytosis with anemia and thrombocytopenia in June 2011. He complained of polymorphous erythema through all the body with herpes and oral ulcer after 4 cycles chemotherapy consisted of 1 cycle CHOP-like [cyclophosphamide (CTX) 750 mg/m^2^ d1, epirubicin 75 mg/m^2^ d1, vindesine (VDS) 3 mg/m^2^ d1, prednisolone (pred) 60 mg/(m^2^ d) d1–5] and 3 cycles FC [fludarabine (Flu) 25 mg/(m^2^ d) d1–3, cyclophosphamide 250 mg/(m^2^ d) d1–3] in September 2012. The skin lesions developed under the combination therapy of antibiotics, anti-viral and antifungal drugs while the therapeutic effect of CLL was partial remission (PR). HE staining, serum antibodies and immunofluorescence were conducted to conduct the diagnosis of PNP (Fig. 1; Table 2). According to the Fig. 1A–C, we found cracks and blisters could be easily seen in sliced side of the epidermis. Acantholism released cells and mixed inflammatory cell infiltrated to cause blisters. It was proved to be moss-like dermatitis changes, especial vice-tumor pemphigus. And IgG and C3 were deposited linearly in the basement membrane (Fig. 2). All above shed a light on the diagnosis of PNP associated CLL. Based on the diagnosis of PNP and the therapeutic effect of CLL, the combination of fresh frozen plasma (FFP) (two units d0), rituximab (375 mg/m^2^, d0), IVIG [0.4 g/(kg d), d1–5] and methylprednisolone[MP, 1 g/(m^2^ d) d1–5] were given for a couple of cycles. The PNP was healed up gradually while the pulmonary infection occurred intermittently. The patient received IVIG (5–10 g per time) intermittently until last follow-up in June 2015.

**Table 2:** Description of PNP in the 6 CLL patients.

Patient	P1	P2	P3	P4	P5	P6
Areas of involvement	Skin, oral	Skin	Skin	Skin	Skin, eye, nose	Skin, mouth, throat
IgG (epidermal side) in serum	Positive	Positive	NA	Positive	Positive	Positive
IgG (dermal side) in serum	Positive	Positive	NA	Positive	Positive	Positive
Aiti-Dsg3 in serum	Positive	Positive	Positive	Positive	Positive	Positive
C3 (epidermal side) in serum	Positive	NA	NA	NA	Positive	NA
C3 (dermal side) in serum	Positive	NA	NA	NA	Positive	NA
Type of skin lesion	Herpes, polymorphous erythema	Erythema, ulceration, exudate	Rash, rupture	Itching, lumps, blisters or ulcers	Red spots and blisters, itching	Massive canker sores, erythema
Histological changes	Moss-like dermatitis changes, especial vice-tumor pemphigus	Epidermal acantholysis and vacuolar interface dermatitis	Lymphatic mononuclear cells infiltrated around the superficial dermis of the dermis, with serous exudation and loosening of the spinous layer in the blister	Moss-like dermatitis changes, especial vice-tumor pemphigus	Acantholysis	Keratinocyte apoptosis
Immunofluorescence	IgG and C3 were deposited linearly in the basement membrane	IgG deposits were found in intercellular and basement membrane	NA	IgG deposits were found in intercellular and basement membrane	NA	NA

Patient 2, was treated with 1 cycle CHOP regimen [cyclophosphamide 750 mg/m^2^ d1, epirubicin 75 mg/m^2^ d1, vindesine 3 mg/m^2^ d1, prednisolone 60 mg/(m^2^ d) d1–5] and 2 cycles of FCM [fludarabine 25 mg/(m^2^ d) d1–3, cyclophosphamide 200 mg/(m^2^ d) d1–3, mitoxantrone 8 mg/m^2^ d1] based on his treatment indications including anemia from September 2004. His CLL status achieved PR and he stopped chemotherapy for 15 months. But he developed night sweat and enlargement of lymphadenopathy gradually. After 2 cycles of high-dose methylprednisolone (HDMP, 1 g/m^2^, d1–5), the patient 2 was proved to develop glomerulosclerosis and PNP according to pathological biopsies. Besides, the sizes of lymph node and the clinical symptoms had no obvious change after anti-infective treatment therapy. Additional therapy of oral methylprednisolone (40 mg/d, about 1 month) brought the patient 2 improvement of clinical symptoms in a short time. The spleen and lymph nodes in superficial region enlarged quickly. Skin biopsy showed epidermal acantholysis and vacuolar interface dermatitis, and IgG deposits were found in intercellular and basement membrane according to immunofluorescence which indicated PNP. The patient 2 suffered an aggressive progression and developed paraneoplastic neurological syndrome in spite of the use of IVIG [0.4 g/(kg d), d1–5] and rituximab (375 mg/m^2^ d0), and finally died of refractory PNP and severe pulmonary infection.

Patient 3 was a 55-year-old male diagnosed CLL in 2002 owing to increased lymphocytes and enlarged lymph nodes, treated with multiple lines chemotherapies including COP [cyclophosphamide 750 mg/m^2^ d1, vindesine sulfate 3 mg/m^2^ d1, hydroprednisone 60 mg/(m^2^ d) d1–5] for 3 cycles and fludarabine[25 mg/(m^2^ d) d1–3] for 6 cycles up to his complete remission of his CLL in the end of 2003. He received FC regimen for 5 cycles, chlorambucil less than a year, CHOP-like regimen for 1 cycle from 2007 due to progression of his CLL. The enlargement of lymph nodes and night sweats indicated the rapid progression of CLL in June 2012. Moreover, the rash scattered in the chest and abdomen. Based on the skin lesions’ histological changes and positive serum antibodies, PNP was taken for consideration. After 5 cycles of combination of FFP (two units d0), rituximab (375 mg/m^2^ d0) and methylprednisolone [1 g/(m^2^ d) d1–5], the skin lesion and symptoms decreased gradually. But his CLL didn’t show any improvement. The patient received an aggressive chemotherapy regimen of Hyper-CVAD-A (CTX 0.3 g/m^2^ q12h d1–3, vincristine 2 mg d4 d11, dexamethasone 40 mg qd d1–4 d11–14, doxorubicin 50 mg/m^2^ d4) combining with bortezomib [1.3 mg/(m^2^ d) d3, d8] and IVIG[0.4 g/(kg d), d1–5] to control his CLL and PNP in a short term. He died in Jul 2013 owing to his CLL disease progression, pulmonary infection and heart failure.

The patient 4 was a 81-year-old female diagnosed with CLL in 2003 without treatment indications. She was treated with FC regimen without definite courses owing to enlarged lymph nodes and achieved PR in 2009, developing skin lesions complained of itching, lumps, blisters or ulcers in the next three years. The superficial lymph nodes decreased after chemotherapy while the skin lesions developed under the treatment of anti-allergic agents. With the pathology of skin lesions and postive serum antibodies (Fig. 1D–F; Table 2), PNP was diagnosed in June 2015. After 3 cycles of combination regimen of FFP (two units d0) rituximab (375 mg/m^2^ d0) and IL-2 (500,000 IU HD qod), the patient recovered from PNP and received IVIG (10 g) each month while her CLL kept stable. The patient was treated with antibiotics for her pulmonary infection in Oct 2017 and her prolymphocytosis percentage in blood smear was up to 20% indicating her CLL was progressed and changed. Even receiving the combination of ibrutinib, antifungal and antibiotics, the patient died of severe oral, maxillofacial and pulmonary infections, bleeding and kidney failure in Nov 2017.

The patient 5 was a 58-year-old female diagnosed as CLL in Oct 2014 with abnormal monoclonal B lymphocytosis based on flow cytometry without treatment indication. Up to Mar 2016, she intermittently took chlorambucil tablets (2 mg po bid) to decrease her lymphocytes proliferation while her lymphocytes count exceeded 100 × 10^9^/L. But red spots and blisters gradually appeared in her bilateral upper and lower limbs accompanied by itching symptoms in May 2017. Even if we gave her advice to receive biopsy in her limbs, she didn’t pay much more attention on her lesions of repeated skin erythema and blisters. Until Sep 2017, she received FCR regimen [Rtx 375 mg/m^2^ d0, fludarabine(Flu) 25 mg/(m^2^ d) d1–3, cyclophosphamide 250 mg/(m^2^ d) d1–3] for 6 cycles(every 28 days a cycle) because of her progressive increase in lymphocyte counts and lymphadenopathy. Her CLL was controlled to PR according to iwCLL while her skin lesions repeatedly appeared, disappeared and partially aggravated in August 2018. She accepted skin biopsy in dermatologic department owing to red spots, blisters and itching while Edema appeared in her eyes and nose. PNP was diagnosed according to anti-Dsg3 positive in serum and histological changes (Table 2). During her hospitalization in August 2018, she received HDMP (1 g/m^2^, d1–3) and immunoglobin transfusion [0.4 g/(kg d), d1–5] based on the diagnosis of PNP. This patient intermittently receives an immunoglobulin infusion (5–10 g each time every month) when there is a significant drop in IgG. The symptoms of itching and erythema have been moderately improved and her CLL disease state assessment reaches Cru (without bone marrow evaluation) in May 2019.

The patient 6 was a 49-year-old male complained of lymphocytosis and lymphadenectasis in Dec 2016 without treatment history. Dexamethasone (10 mg qd × 7ds) was given to the patient as pretreatment in June 2017 to control his dyspnea caused by increased lymphocytes (37 × 10^9^/L) and enlarged lymph nodes. During the pretreatment, the patient developed massive canker sores which was progressive existence and refractory to antibiotics and antifungal drugs while his dyspnea developed intermittently. The pathology of the oral mucosa lesion and serum examination were proved to be PNP (Table 2). With the combination therapy of HDMP [MP 1 g/(m^2^ d), d1–5] and IVIG[0.4 g/(kg d), d1–5], the oral lesion recovered gradually. The 6 cycles of R-COP[RTX 375 mg/m^2^ d0, CTX 750 mg/m^2^ d1, VDS 3 mg/m^2^, pred 60 mg/(m^2^ d) d1–5] were given to the patient to treat CLL with a PR response while oral lesion presented intermittently. The patient changed to take ibrutinib (420 mg/d) combining with dosage-adjusted prednisone acetate from June 2018 to now to control his CLL disease at a Cru (without bone marrow evaluation) stage and recovered from his PNP.

Totally, 5 cases of them received fludarabine-based regimen before the development of PNP except case 6. Although high doses of glucocorticoid were given to all 6 patients at the time of PNP suffered, the outcomes were presented in various way. Patient 2 died of the refractory PNP and the secondary pulmonary infection, patient 3 died of his CLL disease progression, pulmonary infection and heart failure, and patient 4 died of severe complex infections especially pulmonary infection and bleeding, the case 5 and 6 are still alive at the recent follow-up.

## Discussion

Paraneoplastic pemphigus, as a rare and high mortality autoimmune blistering disease accompanied by both benign and malignant neoplasms, was first described by Anhalt et al. in 1990. The relatively frequently reported associated hematological malignancies are B-cell lymphoma, Castleman’s disease, CLL and Waldenström’s macroglobulinemia. Autoimmune diseases especially hemocytopenia are frequently associated with CLL. Patients with CLL/SLL have a 5–10% risk of developing autoimmune complications. It can develop at any stage in the process of CLL^12^. PNP most often occurs in cancer patients aged from 45 to 70 years old, while it can also appear in children and adolescents. Different from other types of pemphigus, PNP can invade not only epithelia in gastrointestinal but also respiratory tract, resulting in florid oral mucosal lesions including extensive polymorphous cutaneous eruption and lung involvement. Kaplan et al.^13^ pointed out that the most severe lesions during PNP occur 2–3 years after NHL diagnosis. Except for the first set of diagnostic criteria of Anhalt et al.^2,13^, there are no clear diagnostic criteria for PNP. The criteria they summarized^2,14^ included characteristic clinical manifestations and histopathology, detection of tissue binding, circulating autoantibodies detected by direct immunofluorescence, indirect immunofluorescence (IIF), and immunoprecipitation studies. Kaplan et al. have revealed that the typical manifestation of painful inflammation in oral mucosa, polymorphous skin eruptions including lichenoid or acantholytic changes in pathology, positive immunofluorescence findings especially the combination of cells and basement membrane, combined with column coexists with serum antibodies of transitional epithelium or lymphoproliferative diseases especially the presence of anti-dsg, desaminoprotin I and II, envoplakin, periplakin, bullous pemphigus antigen 1 and Plectin antibody^11^. The appearance of skin lesions is diverse and has five changes: pemphigoid-like, bullous pemphigoid-like, erythema-like, graft-versus-host disease, lichen planus-like^15^. Bronchiolitis obliterans, as a serious life-threatening complication of PNP, is still unknown in the occurrence frequency and pathological mechanisms, although it was reported in several researches at a lower proportion^16,17^ and was irreversible with aggressive therapy^4,18,19^.

PNP was thought to be a severe autoimmune reaction resulting in the deposition of auto-antibodies in epithelium. It was identified as a distinct disease from the following five aspects: (i) painful mucosal erosions and polymorphous skin eruption in neoplasia, (ii) histological changes (acantholysis, keratinocyte necrosis, interface dermatitis); (iii) DIF showing abnormal deposition of IgG and complement in intercellular substance and basement membrane zone, (iv) IIF with the same deposition as for DIF, in skin or mucosa including simple, columnar, and transitional epithelium, (v) demonstration of serum antibodies through immunoprecipitation of a complex of four keratinocyte proteins (250, 230, 210, 190 kd)^13^. The developing concept of paraneoplastic autoimmune multi-organ syndromes has been proposed due to its clinical individuation and deposition of auto-antibodies could not only be found existed in the skin and mucous membranes but also in multiple internal organs^20^. Skin lesions can be involved in up to 25% of patients with CLL while the more common secondary lesions are presented as purpura, pruritus, urticaria, erythroderma, cutaneous vasculitis, pyoderma gangrenosum, sweet’s syndrome and so on. Above all, erythema multiforme is proved to be a poor prognosis of the disease^21^. Oral mucosa ulceration as the characteristic manifestation should be paid much more attention on to distinguish the life-threatened PNP from the other skin lesions complications of CLL. The possible antibody-mediated pathologic mechanisms in PNP associated neoplasias are listed as follows: Tumor-induced production of autoantibodies targeting epithelial proteins; Cross-reactivity of neoplasm and epithelial antigens; Elevated IL-6 leading to abnormal B-cell differentiation and auto-immunoglobulin production; Epitope spreading (interface dermatitis induced by the neoplasia exposes epidermal epitopes with autoantibody production against multiple epidermal proteins)^3,22^. The stubborn oral ulcerations used to present drug-resistant to conventional treatments. There is no standard regimen for PNP owing to its rarity and severity, diversity of clinical manifestations and tumor heterogeneity. Besides, PNP management involves a complex and multidisciplinary approach aiming to treat the associated hematologic neoplasia and the mucocutaneous lesions. High-dose corticosteroids including prednisolone are still recommended as the first line therapy^23,24^. Corticosteroids merely improve the dermal lesion, while mucosal involvement is not generally improved by steroids^25^, which is consistent with our patients’ symptoms. It is reported that other drugs, including cyclophosphamide, intravenous immunoglobulin (IVIG), Rituximab^23^ associated with corticosteroids show good efficacy and safety in specific patients^22^. IVIG is a good choice to counteract and decrease circulating pathogenic auto-antibodies, and to modulate cytokines, complements and lymphocytes^26^. Recalcitrant mucous involvement can be treated with a combination of adjuvant immunosuppressive agents, immunobiologics and corticosteroids. Considering the relapsed/refractory CLL, fresh frozen plasma was added to enhancing the action of rituximab due to its role in a source of complement^27^.

In our research, 4 of the 6 cases were male even if female gender showed much more tendency to autoimmune diseases. Of note, two-thirds of them were diagnosed as Binet C and Rai III or IV. The development of CLL-associated PNP showed less links with CLL-IPI risk even if low risk CLL-IPI patient could develop PNP. Besides, we found two-thirds developed PNP during their progression of CLL and 5 of the 6 developed PNP after receiving fludarabine-based chemotherapy even if many reports revealed no relationship between PNP and the primary malignant neoplasm in sequence of PNP or neoplasm regardless of progression or remission of CLL. Except the case 6, all cases had skin lesions at different degree while case 1 and case 2 suffered more seriously skin lesions in pathology. Moreover, half of the 6 cases proved to experience repeated oral painful mucosal erosions. Fludarabine use is associated with the occurrence of PNP, suggesting the altered immunity led by fludarabine use may triggered PNP development, just like fludarabine associated autoimmune hemolytic anemia (AIHA). Functional deficits in CD4^+^ CD25^+^ FOXP3^+^ Treg cells after fludarabine treatment are one of the risk factors for autoimmune diseases in CLL patients. Interleukin-2, a cytokine that promotes Treg survival and function, could be beneficial for patients to recover their Tregs in specific population including autoimmune diseases^28,29^. However, whether IL-2 can be suggested as an option for PNP needs much more exploration while our patient 4 was treated with FFP and rituximab which may contribute much more to PNP. Furthermore, we found the primary CLL showed no obvious correlation with control of PNP in the patient 2 which was consistent in many reported cases^21,30^, while the patient 6 diagnosed with Rai I recovered much more quickly from PNP than others. Besides, the development of PNP during the treatments including FC and HDMP with the control of CLL equally remind us that the indolent PNP seems have no relationship with the stage and state of the primary neoplasm^31^.

PNP is often reported resistant to conventional immunosuppressive treatments and constantly leads to death caused by infectious complications and respiratory failure due to its abnormal deposition of auto-antibodies and complements in tracheas. The development of bronchiolitis obliterans was thought to be a severe high-mortality complication of PNP. Similarly, 5 of 6 cases developed various degree of pulmonary infection after the diagnosis of PNP. The common cause of the three dead cases was pulmonary infection. Their diagnosis of lung infection were identified by CT scans and sputum bacteriology examination. Pulmonary symptoms included cough, sputum, fever, auscultation of wet rales. It is a pity that our patients didn’t receive pneumocystis jiroveci pneumonia (PJP) prophylaxis before their treatment regimens including fludarabine. Pulmonary PNP may be existed with lung infection while pathological examination of bronchoalveolar lavage fluid is the most valuable evidence. In our research, 4 of 6 patients benefited from treatments after they received chemoimmunotherapeutic regimen combined of rituximab and HDMP and FFP. Long-term and insistent application of rituximab will produce curative effect. Meanwhile, addition of FFP might offer sufficient complements especially in synergistic effect with rituximab by enhancing antibody depended cell-mediated cytotoxicity during the course of diseases. Besides, 2 of the 6 patients received ibrutinib therapy and the last one took the drug to control his CLL and PNP. Bruton's tyrosine kinase is a kind of Tec family kinase with a well-defined role in the B cell receptor and Fcγ receptor signaling pathways. Ibrutinib, as a classical BTK inhibitor, makes it a uniquely attractive target for a safe and efficacious treatment of autoimmune diseases especially in CLL patients. It is reported by Ito et al. in 2018 that the combination of ibrutinib and rituximab was effective against CLL/SLL-associated PNP, while PNP lesions did not improve with ibrutinib monotherapy^32^. And the last patient in our research took ibrutinib to control his CLL and PNP.

In summary, the above 6 patients of CLL companied with PNP showed favorable outcome than the other cases reported^4,31^, which might be benefit from the combination of FFP, rituximab, standard dose methylprednisolone and ibrutinib according to our speculation.

## Conclusion

Above all, CLL accompanied with PNP as a rare and fetal disease, is resistant to treatment resulting in a highly mortality rate over 75%. Patients with Binet C and previous treatment with fludarabine based chemotherapy are more likely to develop PNP. Prognosis of PNP is heterogeneous while some patients progressed rapidly. And the main cause of death in PNP is pulmonary infection. A surgical cure can be a better choice to treat an operable PNP. In most cases, symptoms in PNP can be well managed by nonsurgical regimens including standard doses of glucocorticoid therapy [prednisone 0.5–1.0 mg/(kg d)], cyclosporin, cyclophosphamide, azathioprine, mycophenolate mofetil and so on. Besides, rituximab at a dose of 375 mg/m^2^ qw × 4w can be used in lymphoma or CLL while it can be used at 1 g once and repeated in 2w in rheumatologic diseases. The rest cycles and dosages can be administered according to clinical response and the rest population of CD20 positive B cells. If the patient showed the poor response to the conventional therapy like high doses of prednisone or the application of rituximab, he might be treated with IVIG combined with rituximab. IVIG can be used at 2 g/kg per month. Except the fact of reducing production of pathogenic autoantibodies, the addition of IVIG doesn’t add additional immunosuppression like other immunosuppressants. Alemtuzumab has been reported sporadically effective against CLL-associated PNP^33,34^. And, ibrutinib as a new kind BTK inhibitor, might shed a bright light. Much more attempts should be made for mechanisms and treatments of this fatal disease.

## Abbreviations

PNP: Paraneoplastic pemphigus
NHL: Non-Hodgkin’s lymphoma
CLL: Chronic lymphocytic leukemia
LPD: Lymphoproliferative diseases
FISH: Fluorescence in situ hybridization
IGHV: Immunoglobulin heavy-chain variable region
LDH: Lactate dehydrogenase
β2-MG: β2-Microglobulin
IVIG: Intravenous immunoglobulin
PR: Partial remission
CHOP: Cyclophosphamide, epirubicin, vindesine and prednisolone
FCM: Fludarabine, cyclophosphamide and mitoxantrone
HDMP: High-dose methylprednisolone
COP: Cyclophosphamide, vindesine sulfate, hydroprednisone
